# Automation of Vapour Pressure Osmometry measurements

**DOI:** 10.1155/S1463924691000329

**Published:** 1991

**Authors:** Y. Belaustegi, M. J. Citores, L. A. Fernández

**Affiliations:** Department of Analytical Chemistry University of The Basque Country P.O. Box 644 Bilbao E-48080 Spain

## Abstract

A program has been developed for the control of Vapour Pressure Osmometry (VPO) measurements. The output signal of a Vapour Pressure Osmometer is read by an A/D converter card installed in one of the expansion slots of a PC microcomputer. The stability of the measurements is checked by analysing the first derivative of the smoothed signals, which is calculated in real time. Sets of repeated measurements are carried out under the supervision of a computer program as a check for their reproducibility. When the set is ended the program calculates the average and its standard deviation.

The program is particularly valuable for evaluating VPO measurements for solution equilibria studies. This interfacing strategy may be applied to any kind of technique in which the time stability of the signals is the basis for defining measurement stability. The automated VPO has other advantages, including low cost and time saving.


Journal of Automatic Chemistry, Vol. 13, No. 5 (September–October 1991), pp. 181–185

### Automation of Vapour Pressure Osmometry measurements

**Y. Belaustegi, M. J. Citores* and L. A. Fernández**

*Department of Analytical Chemistry, University of The Basque Country, P.O. Box 644, E-48080 Bilbao, Spain*

*A program has been developed for the control of Vapour Pressure Osmometry (VPO) measurements. The output signal of a Vapour Pressure Osmometer is read by an A/D converter card installed in one of the expansion slots of a PC microcomputer. The stability of the measurements is checked by analysing the first derivative of the smoothed signals, which is calculated in real time. Sets of repeated measurements are carried out under the supervision of a computer program as a check for their reproducibility. When the set is ended the program calculates the average and its standard deviation.*

*The program is particularly valuable for evaluating VPO measurements for solution equilibria studies. This interfacing strategy may be applied to any kind of technique in which the time-stability of the signals is the basis for defining measurement stability. The automated VPO has other advantages, including low cost and time saving.*

### Introduction

Vapour Pressure Osmometry (VPO) is a useful technique for the determination of molecular weights in aqueous or organic solvents, as well as for the determination of the total osmolality of biological solutions (Bonnar *et al.* [1]). It also has the advantage of allowing the measurement of high viscosity and high osmolal concentration solutions. As a rule, molecular weights in organic solutions ranging from 40 to 35 000 g/mol can be determined.

In this technique, two paired thermistors are located in a cell saturated with solvent vapour. The cell temperature is electronically kept constant within ±0·001 °C. The solvent drops, suspended on both thermistors, give a zero temperature difference. After exchanging one of the solvent drops for a solution drop, condensation of solvent vapour in the solution drop results due to the lower vapour pressure of the solution. The heat released by condensation increases the temperature of the solution drop, thus increasing the vapour pressure. The solvent vapour condensation stops when the vapour pressure of the solution, due to the higher temperature, is equal to the vapour pressure of the pure solvent within the cell. The resulting temperature difference between both thermistors is proportional to the osmolal concentration (Aurrekoetxea [2]).

In the context of solution chemistry this technique can be employed for the determination of the aggregation equilibria of many compounds, such as long chain alkylammonium salts dissolved in organic diluents (Markovits *et al.* [3], Orlandini *et al.* [4]).

However, the output signal of most of the commercially available instruments must be read with a recorder. Thus, it is often difficult to ascertain the stability of the readings with sufficient precision for solution equilibria studies. For these reasons, and in connection with the aggregation studies of alkylammonium salts being carried out in the authors' laboratory (Belaustegi [5]), these instruments were interfaced with microcomputers.

### Experimental

### *Apparatus*

The automation and control of VPO measurements using on-line systems requires three basic elements. First, the measuring instrument; in this case, the Vapour Pressure Osmometer. Second, a control element to send the necessary orders and to receive and store the information from the instrument. This task is usually done by a microcomputer. Finally, a communication link, or interface, between the controller and the instrument is also needed. The osmometer is not an intelligent instrument because it does not have an interface and its functioning cannot be directly controlled by a computer. For this reason a previous analogue-to-digital conversion (A/D) of the signal that comes from the thermistor is needed. The A/D converter is used also because it is a very simple and economic way to acquire the information contained in the signal.

The following elements were used for the automated VPO measurements:

(1) A KNAUER Vapour Pressure Osmometer.
(2) A compatible PC/XT COMTRADE microcomputer with 640 Kb RAM working at 8 MHz.
(3) A 12-bit, 16-channel A/D converter card (PC-LABCARD PCL 812) prepared to read analogue signals within ±1 DCV which was set up in one of the expansion slots of the microcomputer. The precision in the conversions is given by:

$$2/(2^{12} - 1) = 4{\cdot}884 \star 10^{-4}\ \mathrm{V} \approx 0{\cdot}49\ \mathrm{mV}.$$

Figure 1 is a schematic representation of the hardware used in the automated VPO measurements.

### *Software*

The automatic system was controlled by a program called OSMIAS. This program was written in compiled BASIC language (Microsoft QuickBASIC 4.0). The measurements are stored in an ASCII file with the following

* Author to whom correspondence should be addressed.

0142–0453/91 $3.00 © 1991 Taylor & Francis Ltd

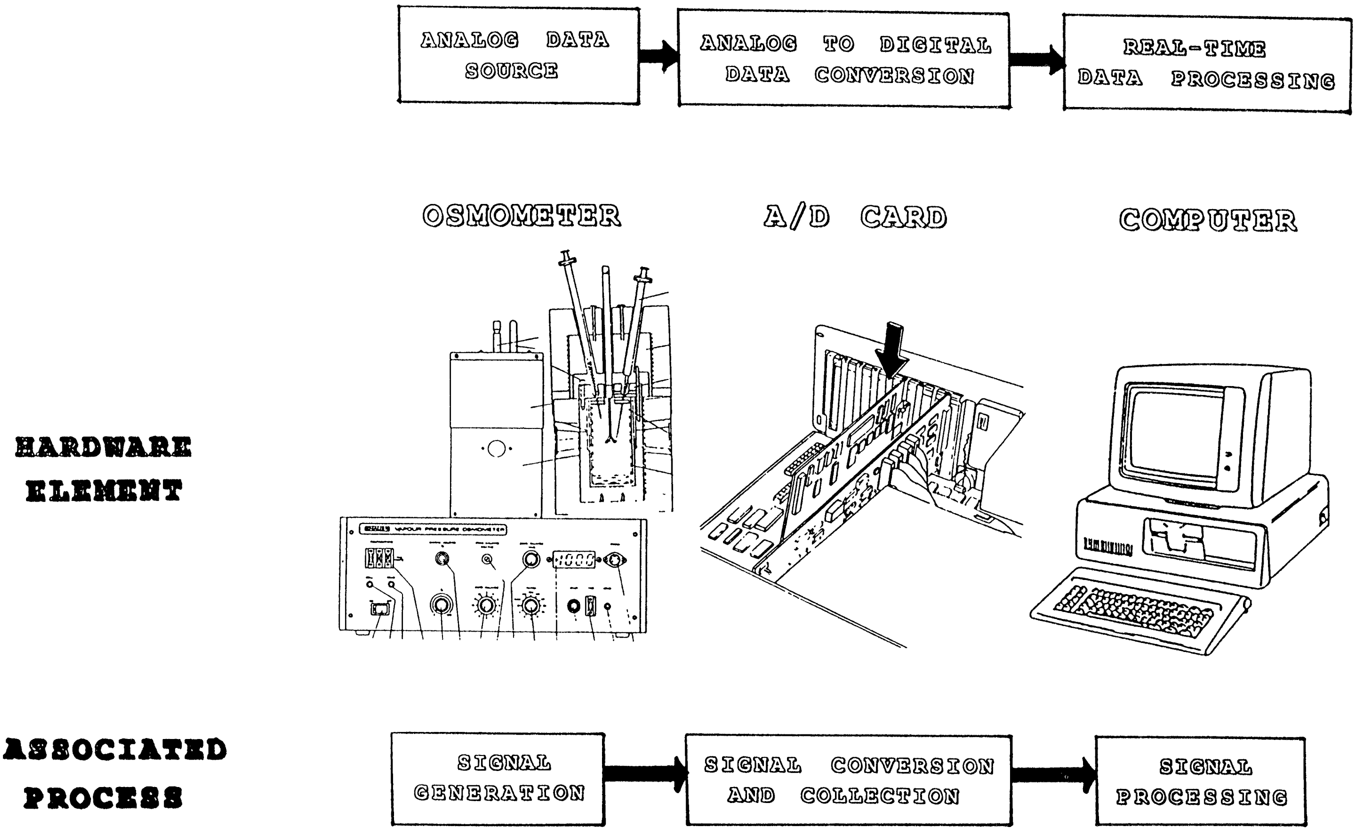

*Figure 1. Schematic representation of the hardware used in the automated VPO measurements.*

structure:

$$2$$
$$(POT_i, TPO_i)_{ni}$$

where:

2 ≡ number of columns in the file
POT and TPO ≡ values of the e.m.f. and time at which the signal from the osmometer's output was collected
i ≡ point index
$n_i$ ≡ total number of points.

*Data acquisition*

An algorithm for the real-time monitoring of the signal from the osmometer was included in the program. Signals are acquired and evaluated through the following sequence of operations:

(1) The A/D conversion of the signal from the osmometer is carried out (starting the moment in which the drops in the thermistor are changed) at short time intervals preset by the user.

(2) From the 18th conversion, the signal values are filtered using a $2^{nd}$ degree Savitzky-Golay algorithm with a 9-point window (Savitzky *et al.* [6]).

(3) From the 20th measurement the first derivative of the signal's filtered values is calculated. For this operation a classic algorithm of finite differences is used.

(4) From that moment the stability of the signal is checked by analysing the first derivative of the smoothed signal. When the variation of this derivative is less than $10^{-3}$ mV, over a certain period of time, the original signal is considered to be constant and the measurement ended.

A flow diagram of this process is presented in figure 2

*Data presentation*

While the signal is being monitored, the plot of the signal's e.m.f. (in Volts) versus the time elapsed from the beginning of the data acquisition (in seconds) is displayed

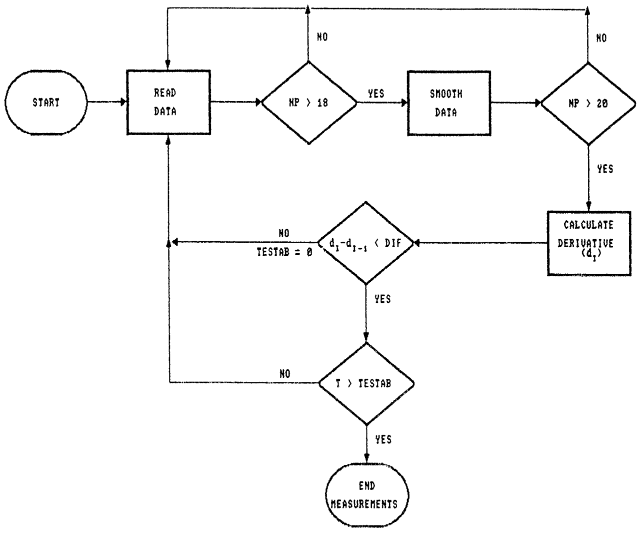

*Figure 2. Flow diagram of the measuring and stability check process.*

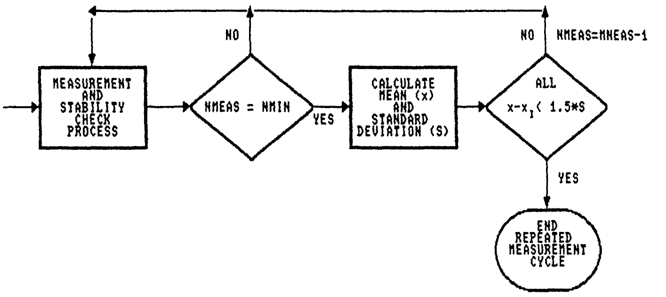

*Figure 3. Flow diagram of the process used to control the reproducibility of a set of VPO measurements.*

on the screen in real time. The following options are also available during signal evaluation:

(1) To re-scale the graph on the screen.
(2) To increase the total monitoring time.
(3) To decrease the total monitoring time.
(4) To stop signal evaluation.
(5) To stop the program.

*Program options*

The control program for the VPO measurements has three main options:

(1) To perform a single measurement.
(2) To perform a whole set of measurements.
(3) To complete a set of measurements previously started.

In each of these options it is necessary to define several parameters before completing the general input of the necessary data to begin the measurements.

An interesting situation is one in which sets of repeated measurements are carried out under the supervision of the program in order to check their reproducibility. When the set of measurements is ended the program calculates the average of the stabilized signal values and the standard deviation. If the deviation of any of the individual measurements is higher than 1·5 times the standard deviation, then the measurements are rejected and repeated. This process is continued until all the measurements fall within the mentioned interval.

A flow diagram of this process is shown in figure 3.

### Results and discussion

The osmometer produces analogue signals that may behave differently depending on such factors as:

(1) The shape and size of the drops in the thermistors
(2) The amount and type of solute in the solution drop
(3) The order in which drops are changed.

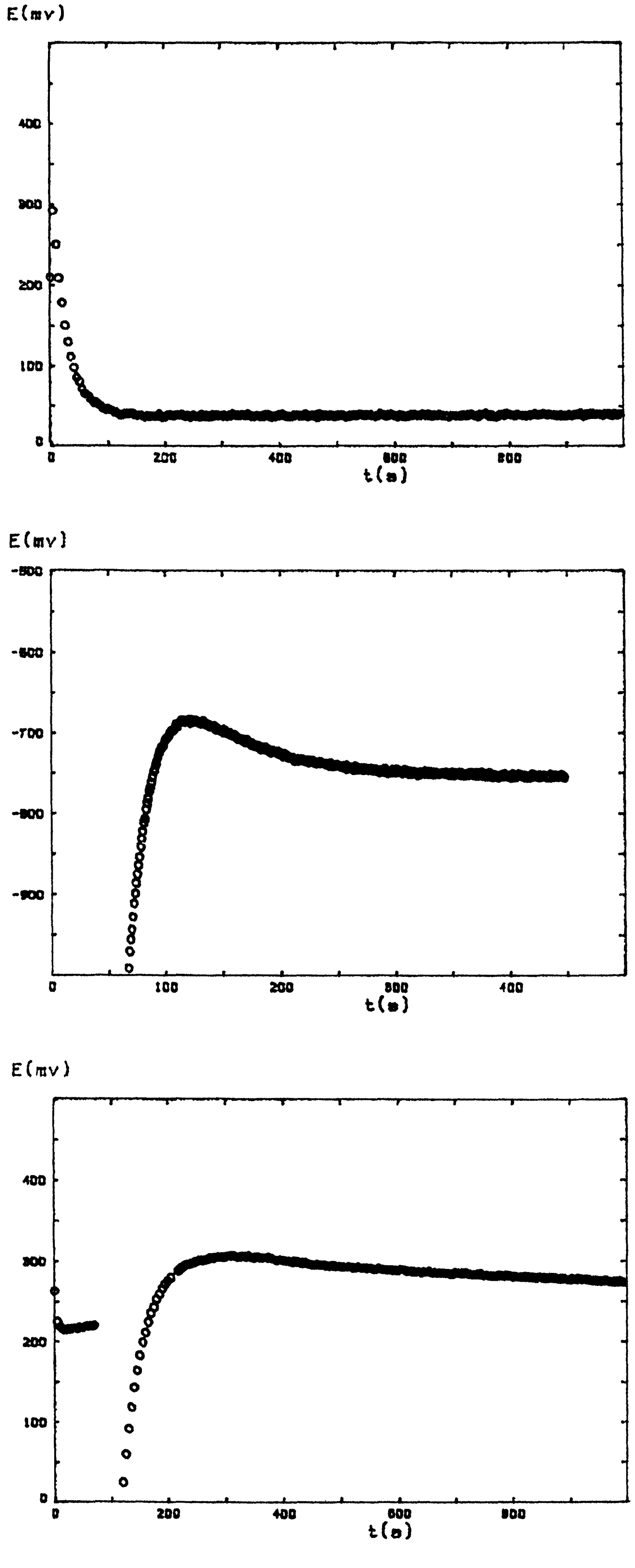

*Figure 4. Different types of VPO signals.*

Figure 4 shows different types of VPO signals. Due to the different shapes of these signals, a general algorithm that is suitable for all of them must be used for their evaluation. The experience gained with the development and use of the program shows that this may be done in the following way.

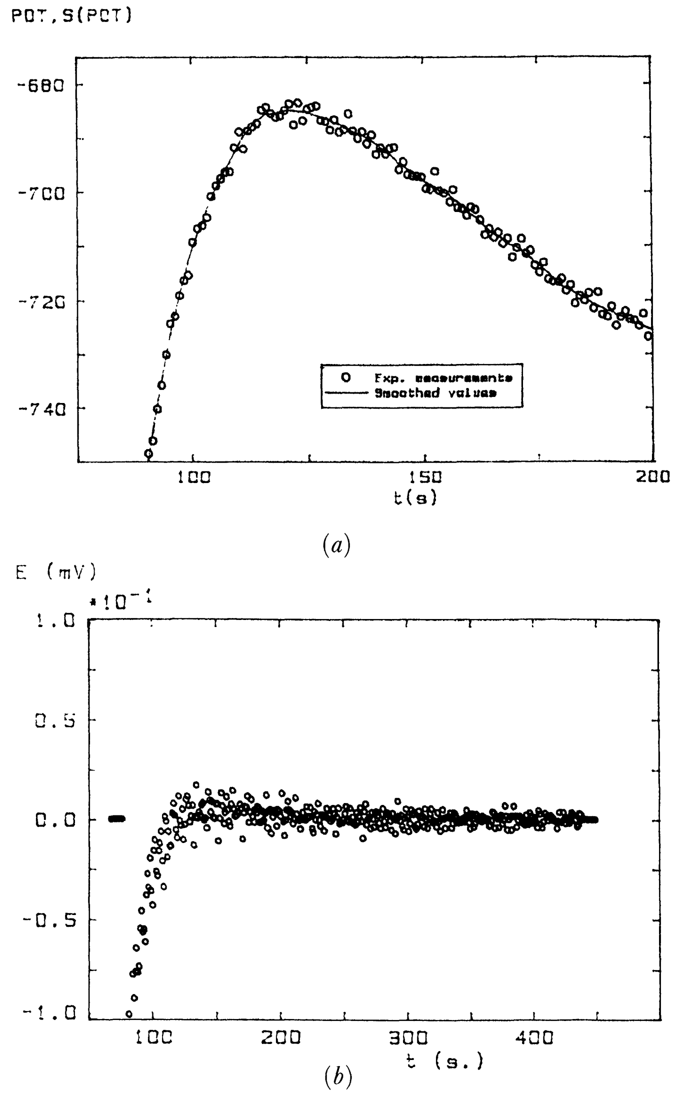

*Figure 5. (a) Typical signal from the osmometer showing the sampling strategy; (b) first derivative of a VPO signal showing the stability.*

*Sampling of the signals*

VPO signals are monitored in real time over a TTOT time interval (20 min)* at DT time intervals (1–2 s). Smoothing of the signal by means of the Savitzky-Golay method is performed in the time between samplings.

*Check for the stability of the measurements*

Signal stability is checked by analysing, also in real-time, the first derivative of the smoothed signal. If the variation in the first derivative is less than DIF ($10^{-3}$ V) during the last TESTAB minutes (3 min) the signal is considered steady and the measurement value is taken as the first which satisfies the stability criterion.

Figure 5 presents a typical signal showing the sampling strategy, together with its first derivative.

* The values within brackets indicate the most commonly used values.

*Check for the reproducibility of a set of measurements*

The following statistical analysis is carried out in order to verify the reproducibility of a set of measurements:

(1) A minimum number NMIN of measurements (4) is performed yielding $X_i$ values.

(2) The average ($X_{ave}$) and standard deviation (S) of these measurements are calculated.

(3) If any of ($X_{ave} - X_i$) < 1·5·S, then that particular $X_i$ value is rejected and the measurement is repeated.

(4) $X_{ave}$ and S are recalculated with the new $X_i$ as in step 2.

(5) The check in step 3 is performed again until all values are accepted.

Figure 6 shows the plot of an acceptable set of measurements according to the statistical criteria used, together with another measurement that is rejected.

This technique has been used to determine the aggregation equilibria in toluene of the following tri-n-laurylammonium (TLA) salts: $TLAHCl$, $TLAHNO_3$, $TLAHClO_4$, $TLAH_2CrO_4$, $TLAH_2SO_4$ and in mixtures $TLAHCl/TLAHNO_3$ and $TLAHCl/TLAHClO_4$ using the data collected making use of this automated system (Belaustegi *et al.* [7]).

### Conclusions

The proposed automation scheme and software for the evaluation of VPO measurements has proved to be adequate for solution equilibria studies. The system has other advantages, for example:

(1) Eliminates the need of an attached recorder.

(2) Allows easy checking of the stability of the measurements.

(3) Allows real time monitoring and treatment of the signals.

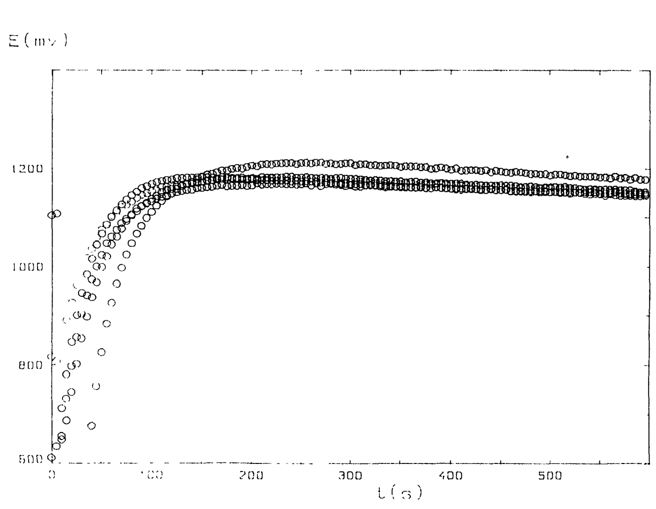

*Figure 6. Plot of an acceptable set of measurements according to the statistical criterion used.*

(4) Saves time, eliminating almost completely the need for human intervention in the measurements.
(5) Allows an improved evaluation of the steady signal values.
(6) Allows data storage for further off-line treatment.
(7) Has a low cost, both in investment and maintenance.
(8) Can be easily adapted to other experimental situations in which the time-stability of the measurements is important.

### References

1. Bonnar, R., Dimbat, H. and Stross, F., *Number Average Molecular Weights* (Interscience Publishers, New York, 1959).
2. Aurrekoetxea, M., B.Sc. Thesis, University of The Basque Country, Bilbao (1980).
3. Markovits, G. and Kertes, A. S., in *Solvent Extraction Chemistry*, Ed. Dyrssen, D., Liljenzin, J. O. and Rydberg, J. (North Holland, Amsterdam, 1967), p. 390.
4. Orlandini, F., Danesi, P. R., Basal, J. and Scibona, G., in *Solvent Extraction Chemistry*, Ed. Dyrssen, D., Liljenzin, J. O. and Rydberg, J. (North Holland, Amsterdam, 1967), p. 408.
5. Belaustegi, Y., B.Sc. Thesis, University of The Basque Country, Bilbao (1990).
6. Savitzky, A. and Golay, M. I. E., *Analytical Chemistry*, **36** (1964), 1627.
7. Belaustegi, Y., Citores, M. J., Etxebarria, N., Olazabal, M. A., Fernández, L. A. and Madariaga, J. M., *Proceedings of the XVII Annual Congress. II. Italian-Spanish Congress on Thermodynamics of Metal Complexes*, Palermo, Italy (1991).

